# Development and Validation of an Age-Specific Lower Extremity Finite Element Model for Simulating Pedestrian Accidents

**DOI:** 10.1155/2018/5906987

**Published:** 2018-03-21

**Authors:** Jing Huang, Yongcheng Long, Yu Yan, Lin Hu

**Affiliations:** ^1^Research Centre of Vehicle and Traffic Safety, State Key Laboratory of Advanced Design and Manufacturing for Vehicle Body, Hunan University, Changsha, China; ^2^State Key Laboratory of Vehicle NVH and Safety Technology, Chongqing, China; ^3^College of Automotive and Mechanical Engineering, Changsha University of Science & Technology, Changsha, China

## Abstract

The objective of the present study is to develop an age-specific lower extremity finite element model for pedestrian accident simulation. Finite element (FE) models have been used as a versatile tool to simulate and understand the pedestrian injury mechanisms and assess injury risk during crashes. However, current computational models only represent certain ages in the population, the age spectrum of the pedestrian victims is very large, and the geometry of anatomical structures and material property of the lower extremities changes with age for adults, which could affect the injury tolerance, especially in at-risk populations such as the elderly. The effects of age on the material mechanical property of bone and soft tissues of the lower extremities as well as the geometry of the long bone were studied. Then an existing 50th percentile male pedestrian lower extremity model was rebuilt to depict lower extremity morphology for 30- to 70-year-old (YO) individuals. A series of PMHS tests were simulated to validate the biofidelity and stability of the created age-specific models and evaluate the lower extremity response. The development of age-specific lower extremity models will lead to an improved understanding of the pedestrian lower extremity injury mechanisms and injury risk prediction for the whole population in vehicle-pedestrian collision accidents.

## 1. Introduction

Pedestrians are road users vulnerable to traffic accidents, who suffer high injury rate and mortality rate. WHO reported that more than one-fifth of the people killed on the world's roads each year are pedestrians [1]; the situation is worse in China, as the road traffic situation in China is more complex than that of developed countries in Europe and America. The vehicle-pedestrian-mixed traffic in most urban and rural roads presents hidden dangers, inhibiting pedestrian safety. The lower extremities and head are the main injury body regions for pedestrians in a vehicle-pedestrian collision accident, accounting for 31.2% and 32.4%, respectively [2]. Compared with head injuries, lower extremity injuries rarely lead directly to fatalities; however, they often cause long-term or life-long disabilities.

Pedestrian injuries are preventable; however, successful interventions to protect pedestrians and promote safe traveling require a better understanding of the injury mechanisms and risk factors for pedestrian crashes. Many researchers established lower extremity models to study its injury mechanisms in pedestrian collision accidents. Zhang et al. [3, 4] improved and verified the material model of the long bone and ligament of the lower extremity based on the THUMS model. Untaroiu et al. [5], using a human body extremity model, combined with an accident reconstruction method, simulated pedestrian lower extremity fractures in collision. Wang et al. [6] used the multibody system and finite element model to study the long bone fracture of the lower limb based on two cases of real pedestrian accidents. Meng et al. [7] established a 6-YO child's lower extremity long bone model and validated this model with a dynamic load three-point bending test, then discussed the effect of elastic modulus on the injury. Shen et al. [8] established a 10-YO child pelvis and lower extremity FE model with growth plates for pedestrian protection. Kong [9] analyzed the pedestrian-vehicle collision accidents in Changsha using statistical methods. Her conclusions noted that children aged 0 to 10 years and middle-aged people over 46 years were more likely than other age groups to suffer higher fatality rates, with death rates increasing with age for people over 46 YO. In literature [10], the data in the American Pedestrian Injury Causation Study (PCDS) database was analyzed statistically; the results showed that the injury risk to the lower extremities of the elderly was 2.44 times higher than that of the young people, and the elderly would suffer more serious injuries and require longer treatment cycles. Hu et al. research results showed that age had an important effect on the injury risk when pedestrians collided with different front-shaped vehicles [11]. All of these research works indicate that age is one of the most important factors affecting the injury risk of pedestrian lower extremities; what is more, the effect of age on injury is nonlinear [12–14].

On the other hand, with the continued rapid growth of the elderly population of adults aged 60+ years, which has increased to 210 million (15.5% of the total population by the end of 2014 [15]), the proportion of the elderly in traffic accidents increased gradually and was as high as 40% [16], according to the statistics of the Ministry of Public Security. Compared with that of young people, the geometry of anatomical structures and material property of the elderly are quite different [17]. With the further development of pedestrian safety research, it is critical to understand the biomechanics change of the human lower extremities with age for pedestrian protection and the establishment of especially vulnerable road user models (such as children, the fifth percentile women, obese people, and the elderly) [18].

The finite element model provides a useful tool to assess injury risk and to study the injury biomechanics, while current models are limited to certain ages in the population. Therefore, it cannot reflect the difference of pedestrian injury at different ages in the accident. The objective of the present study is to investigate the geometric changes and material property changes with aging for pedestrian lower extremities and to develop and validate the age-specific FE models of pedestrian lower extremities to accurately model lower extremity morphology and material property for ages between 30 and 70 years.

## 2. Materials and Methods

### 2.1. Geometric Changes with Aging

In total, 320 femoral and 99 tibial midshafts derived from individuals aged 21–99 years were examined and measured [19–21]; the aim was to determine the age-related changes in the structure of the human long bone. The geometry of the long bone cross-section naturally changed with aging, and the change is associated with marrow cavity area changes.

**Figure 1:**
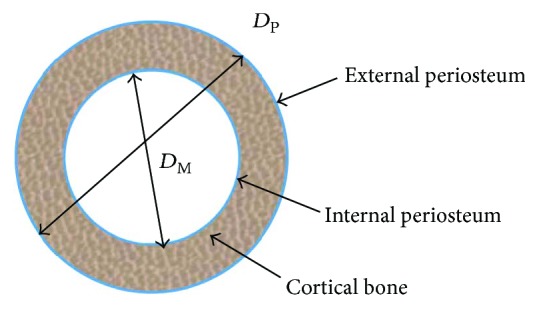
Idealized long bone section.

Figure 1 shows the idealized long bone cross-section. The total area (TA), medullary area (MA), and cortical area (CA) are calculated in (1), (2), and (3), respectively, together with external diameter (*D*_P_) and internal diameter (*D*_M_).

(1) $$\begin{array}{c} TA=\frac{\pi D_{P}^{2}}{4}, \end{array}$$

(2) $$\begin{array}{c} MA=\frac{\pi D_{M}^{2}}{4}, \end{array}$$

(3) $$\begin{array}{c} CA=TA-MA. \end{array}$$

**Figure 2:**
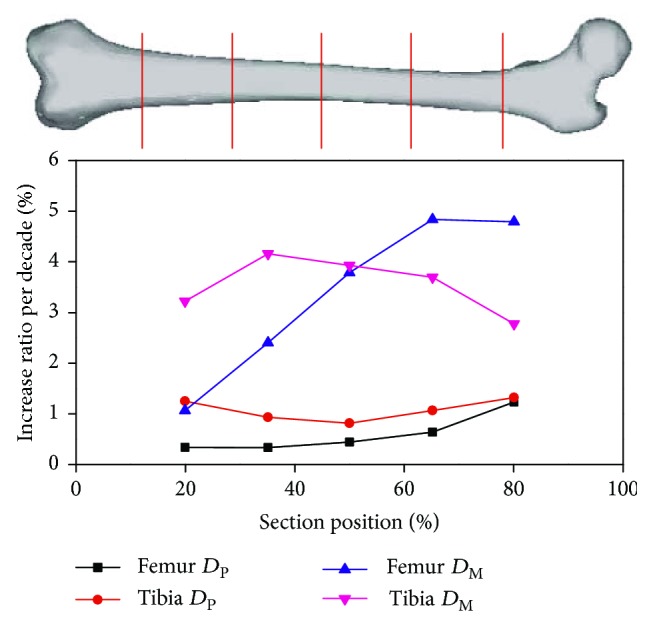
Variation of geometric dimensions of long bone cross-section (male).

According to the values of TA, MA, and CA of different ages obtained by Ruff et al. [21], the increase ratio per decade for *D*_P_ and *D*_M_ of the adult male long bone at 5 cross-sections can be calculated, as shown in Figure 2; then the *D*_P_ and *D*_M_ of a certain age can be scaled from the basic model via the corresponding scale ratio. As for the fibula, due to the shortage of anthropometry data related with age, its geometric change with age is assumed to be the same with the tibia.

Taking a 70-YO adult male for example, the basic lower extremity model used in this manuscript is derived from an adult male aging 26 years, according to Figure 2; the scaling ratios of the *D_P_* and *D*_M_ of the femur and tibia are shown in Table 1; then the *D*_P_ and *D*_M_ of the 70-YO adult can be scaled from the basic model via the corresponding scale ratio at five cross-sections.

**Figure 3:**
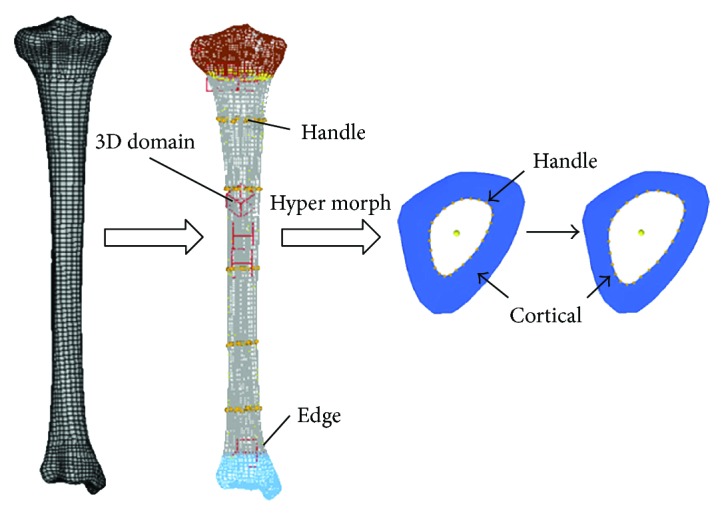
Changes of skeletal mesh with age in finite element model.

Geometric changes of anatomical structures with aging were implemented by changing the long bone cross-section—model morphing can be used to generate models of all ages accurately and efficiently [22]. The HyperMorph module of HyperMesh software is used to rebuild an existing 50th percentile male adult FE model to obtain the lower extremity models in the full spectrum of ages, as shown in Figure 3. 3D adjust domain was established in 5 cross-sections in long bone meshes firstly, and the control point handle of the experimental point was setup in a corresponding position based on previous geometric study results. Then the location of the control point could be adjusted manually to complete the update of lone bone meshes to get the age-specific models.

### 2.2. Material Property Changes with Aging

#### 2.2.1. Long Bone

The cancellous bone of the lower extremity is a kind of porous structure composed of irregularly arranged trabecular bones; its mechanical properties are similar to foamed aluminum. When compressed, there is a significant elastic phase and the stress is nearly unchanged after the yield point; the limit stress is almost equal to the yield stress. The material properties of the cancellous bone showed obvious changes with aging because of the loss of calcification and fibrosis. Its material properties can be simulated by the material model of dynamic elastoplastic (^∗^ MAT_PLASTIC_KINEMATIC), and the ultimate stress is set to 13.4% according to the research results of literature [23].

The quasistatic compression test data of the cancellous bone from the ages of 16 to 83 years [23] were subjected to quadratic polynomial fitting. The results showed that the mechanical properties of cancellous bone increased from 20 to 40 years, while there is a sudden drop after 40 years. The correlation between age and elastic modulus and ultimate stress is developed as

(4) $$\begin{array}{c} Elastic\;modulus=473.3+14.99\;age-0.19\;{age}^{2},\\ Ultimate\;stress=8.94+0.13\;age-0.002\;{age}^{2}. \end{array}$$

The material properties of the cortical bone are simulated by the material model of isotropic elastoplastic (^∗^ MAT_PIECEWISE_LINEAR_PLASTICITY); as the slope of its stress-strain curve in the plastic stage remains unchanged [24], it can be assumed that the tangent modulus in the plastic stage will not change with aging. If the strain of meshes rose to the failure strain, the fracture would occur and be simulated by mesh deletion. The Cowper-Symonds method is used to simulate the effect of strain rate on the material properties, with the yield stress scaling equation shown in

(5) $$\begin{array}{c} \sigma \left(\varepsilon ,\dot{\varepsilon }\right)=\sigma_{0}\left(\varepsilon \right)\left[1+{\left(\frac{\dot{\varepsilon }}{C}\right)}^{\left(1/p\right)}\right], \end{array}$$

where *σ*_0_(*ε*) is the initial yield stress, while $\dot{\varepsilon }$ is the strain rate. *C* and *P* are the strain rate transformation parameters. In this paper, *C* is 360.5 and *P* is 3.6.

The elastic modulus, ultimate stress, and failure strain of the cortical bone of different age groups are obtained by regression analysis of corresponding test data in literatures [25, 26] and [27]. The correlation between age and elastic modulus, ultimate stress, and failure strain is developed as presented in (6), (7), and (8), respectively.

(6) $$\begin{array}{c} Elastic\;modulus=18.01-0.059\;age, \end{array}$$

(7) $$\begin{array}{c} Ultimate\;stress=130.8-0.52\;age, \end{array}$$

(8) $$\begin{array}{c} Failure\;strain=4.23-0.033\;age. \end{array}$$

Based on the research results of the literature [28, 29], the change of material property of the cancellous bone and cortical bone is assumed to be the same for lower limb long bones, as they undergo the same changes with age. The material property parameters of the cortical bone and cancellous bone of the long bones for different ages can be calculated according to the above fitting formulas and corresponding scaling coefficients.  Table 2 shows the lower extremity long bone material properties of ages 30 years and 70 years for example.

#### 2.2.2. Ligaments

The ligaments in the knee are connected to the bones, which stabilize and restrict the movement of the knee, including the patellar ligament, meniscofemoral ligament, medial collateral ligament (MCL), lateral collateral ligament (LCL), anterior cruciate ligament (ACL), and posterior cruciate ligament (PCL). The diameter of collagen fibers decreased, while the fiber content increased with aging. For example, the maximum fiber diameter is 180 nm when a man is 15–19 YO and reduced to 110 nm after 60 YO [30, 31]. The change of collagen fibrils will affect the mechanical properties of the ligament; the ultimate tension, especially, will decline with aging [31].

In the present study, the ligaments are simulated by the solid elements to accurately model the geometrical shape of each ligament and their contact with the surrounding tissue. The hyperelastic material constitutive model (^∗^ MAT_SOFT_TISSUE) is used to simulate its mechanical properties [32], and the failure of the first-order principal strain is defined for the elements, with the laceration of ligament simulated by element deletion.

**Figure 4:**
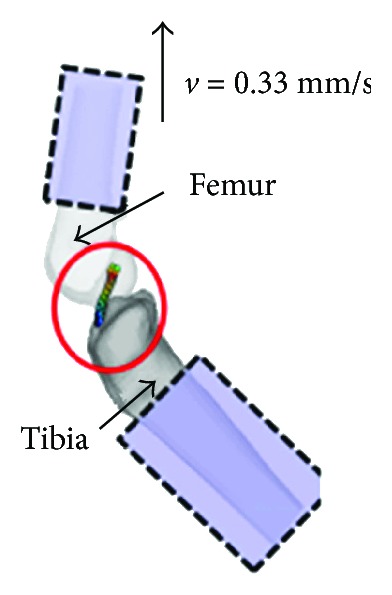
Knee ligament simulation model.

The experiments of the knee joint ligament carried out by Woo et al. [33] were simulated; the simulation model is shown in Figure 4. The ligament properties for different ages can be obtained by parameter computational inverse based on the ligament tensile test curves.

Table 3 shows the knee joint ligament material properties of individuals aging 30 to 70 years; for example, C1, C3, C4, and C5 are the parameters of the material model.

### 2.3. Development of the Age-Specific Lower Extremity FE Model

**Figure 5:**
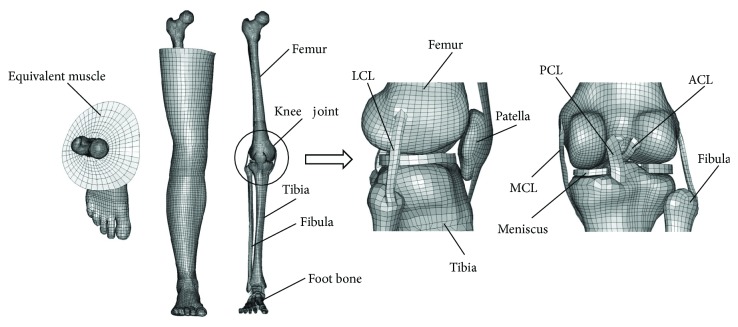
Finite element model of human lower extremity with age characteristics.

The baseline pedestrian lower extremity model is derived from the Global Human Body Models Consortium (GHBMC) average male occupant model. The GHBMC is representative of a 50th percentile male adult and was based on medical images of a 26 YO individual. The lower extremity model includes the long bone, muscle, ligament, skin, and other tissues. The cortical bone and cancellous bone of the long bone shaft are modeled using hexahedral elements. The cortical bone covering the long bone ends is modeled using quadrilateral shell elements. Muscle and skin are modeled using the solid element and shell element, respectively. Ligaments are modeled using the solid element and one-dimensional beam element together. The baseline model is adjusted according to the pedestrian's standing posture. Then the previous research results of the geometric changes and material property changes with aging are applied to build the age-specific lower extremity FE models—including the adjustment of the material properties and the geometry morphing of the femur, tibia, and fibula, as shown in Figure 5. Then two FE models of the pedestrian lower extremity of typical ages 30 and 70 years are established to investigate the effect of age on injury risk. The selection of 30- and 70-YO models was based on a previous recommendation that defined a young adult group between 16 and 35 YO and elderly group as 66 YO and older [34] .

### 2.4. Model Validation

A series of cadaver test data were used to validate the biofidelity and stability of age-specific pedestrian lower extremity FE models, as shown in Table 4. These validation tests were simulated in LS-DYNA software according to the published test information.

#### 2.4.1. Validation at the Component Level

**Figure 6:**
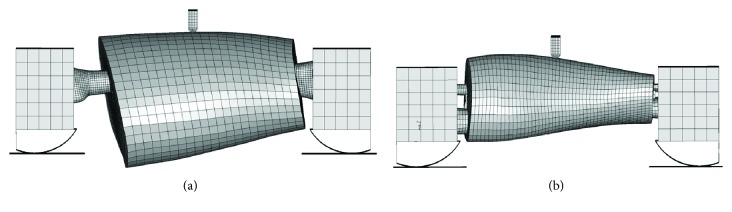
Thigh and calf three-point bending test model: (a) thigh and (b) calf.

In Kerrigan's test [35], the thigh and calf were extracted from PMHS. The muscle tissues of two ends were removed, and the distal and proximal ends of the femur and tibia were potted in cups and fixed with polyurethane. An impactor driven by a universe machine loaded the thigh and calf at the middle-shaft location at the speed of 1.5 m/s to simulate the loading condition of pedestrian lower extremities in a vehicle-pedestrian collision accident. Then two age-specific FE models of the pedestrian lower extremities of typical ages 30 and 70 YO were used to simulate the same tests with the same experiment settings and boundaries. The finite element models are shown in Figure 6.

**Figure 7:**
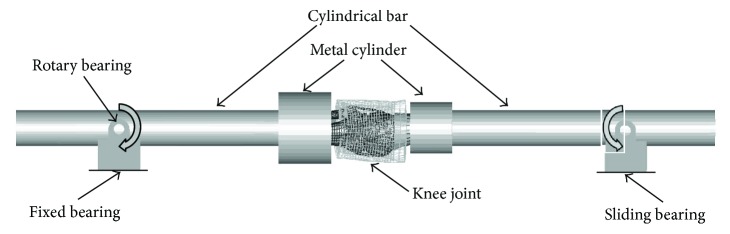
Four-point bending test device and finite element model.

Ligament failure caused by lateral bending is a common knee injury for pedestrian during vehicle-pedestrian collision accident. Kerrigan et al. [35] and Bose et al. [37] designed a dynamic four-point bending test to estimate knee tolerance.  Figure 7 illustrates the test principle: isolated knee parts were potted in specific cups that rotated around support joints during tests. While the distal support connected to the tibia was fixed, the proximal support connected to the femur was allowed to move horizontally. The angular speed of the knee was about 1 deg/ms during tests to simulate the knee-bending load when pedestrian crashed at a speed of 40 Km/h, and the bending moment was measured by a load cell connected to the femur extension bar. Corresponding simulation models were built to perform the same tests in ls-dyna, and then the simulation results were compared with the test data.

#### 2.4.2. Validation at the Lower Extremity Level

To evaluate the whole lower extremity response, 2 loading cases, bending and shear, were simulated to assess the importance of geometric and material property changes with aging.

**Figure 8:**
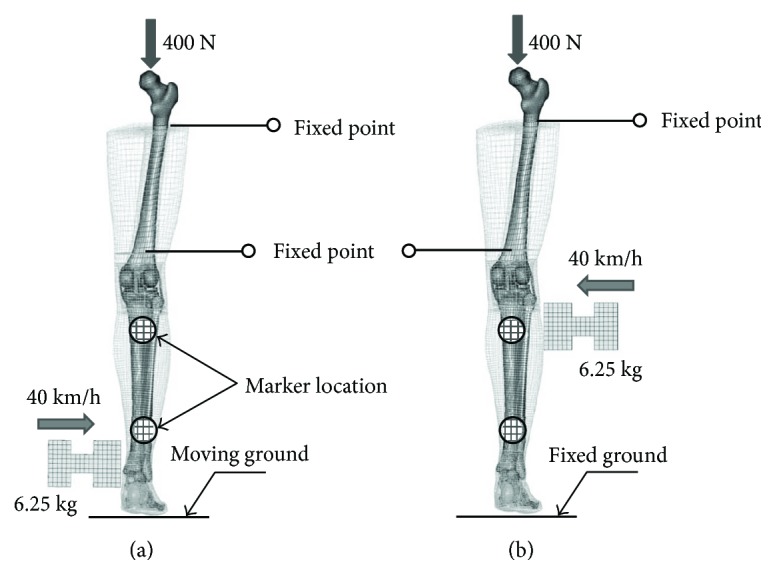
Lower extremity bending (a) and shear (b) simulation model.

According to the tests of Kajzer et al. [38], as shown in Figure 8, the lower extremity was extracted from PMHS on the hip joint and fixed flat on a board to maintain stability. The proximal of the femur was fixed with screws, while the distal of the femur was fixed with a fixed plate to limit its horizontal movement. The force sensor would calculate the bending moment of the knee joint. A force of 400 N was loaded at the hip to simulate the load received by the lower extremities when standing. The bending and shear impact load was conducted at 40 km/h with a 6.25 kg I-shaped impactor striking the ankle joint and the knee joint, respectively. The impactor was wrapped with a foam of 100 mm × 120 mm × 50 mm at the front.

## 3. Results and Discussion

**Figure 9:**
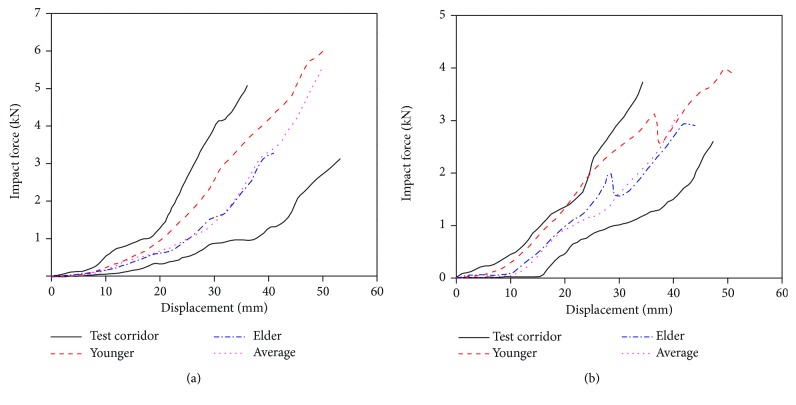
Force displacement curves of the impactor in three-point bending simulation of the (a) thigh and (b) calf.

The force displacement curves of the impactor in thigh and calf three-point bending simulations are shown in Figure 9. Both the simulation results of young (30 YO) and elderly (70 YO) are in the test corridor and consistent with the experimental results, though different from each other. This indicates that the response of the young and the elderly is much different. The impactor force rises slowly initially but is followed by a sharp increase. This is because the impactor makes contact with the skin, muscle, and other soft tissues first, and when the femur begins to bend to deform, the force increases.

**Figure 10:**
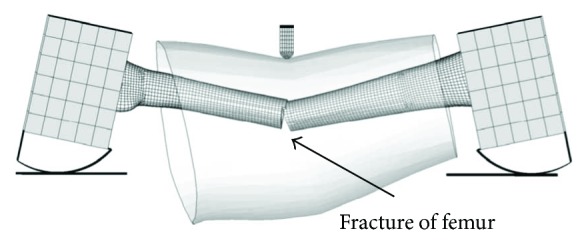
Femur fracture location in thigh three-point bending simulation.

In thigh three-point bending simulation, the femoral fracture occurred in both cases of the young (30 YO) and the elderly (70 YO), as shown in Figure 10. For the elderly, the femur fracture occurred when the displacement of the impactor is 40 mm with the impact force 3.2 kN, while the corresponding data of the young is 50 mm and 6.1 kN.

**Figure 11:**
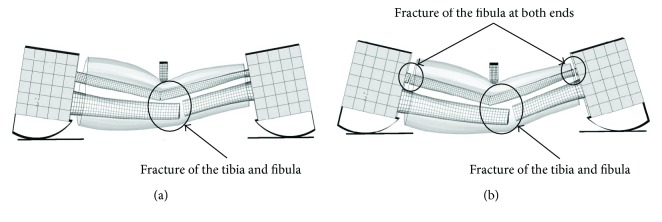
Tibia and fibula fracture location in calf three-point bending simulation: (a) the young and (b) the elderly.

In calf bending simulation, both the tibia and fibula are fractured, while the fracture locations are different, as shown in Figure 11. The elderly's fibula was fractured at both ends and the middle shaft, but the young's fibula was only fractured in the middle. For the elderly, the fibula fracture occurred when the displacement of the impactor is 29 mm, while the young is 36 mm, and the curve showed an obvious decline when the fibula fractured. After the fibula fracture, the impactor continued to load on the tibia, and both the young and the elderly suffered tibia fracture subsequently when the displacement was 50 mm and 42 mm, respectively.

**Figure 12:**
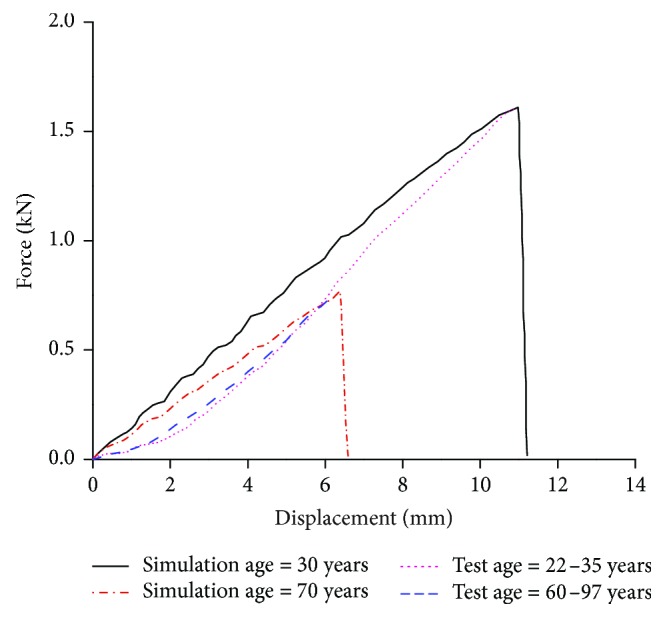
Ligament ACL displacement force curve comparison between experiment and simulation.

The comparison among the ligament displacement force is shown in Figure 12 in terms of results from the simulation of the models and experimental data of Woo et al. [33] in the tensile tests of the femur-ACL-tibia complex. Both the simulation curves for the young and the elderly are well aligned with the experimental results. It is believed that the material property parameters of the ligaments for different ages are reasonable and can reflect the ligament injury at different ages.

**Figure 13:**
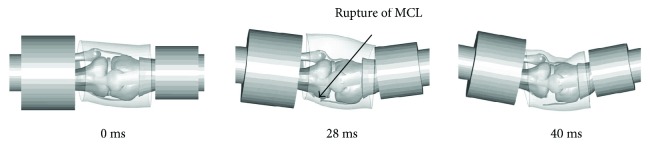
Knee four-point bending simulation process.

Figure 13 shows the results of a knee joint four-point bending simulation of the young. The medial collateral ligament (MCL) is completely ruptured at 28 ms near the tibia junction, which coincides with the test results performed by Kerrigan et al. [35] and Bose et al. [37].

**Figure 14:**
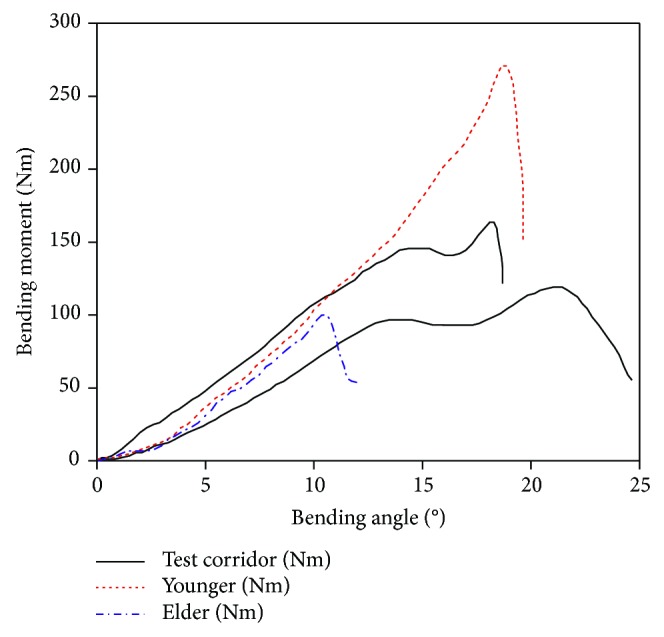
Curve of bending angle and bending moment of knees in four-point bending simulation.

The bending-angle-to-bending-moment curves of the knee joint are shown in Figure 14.

The simulation results of the elderly are in the test corridor, while the peak of the young is outside the corridor. This may be due to the ages of the PMHS, as they are between 44 YO and 80 YO—therefore, it is reasonable that the peak of the young (30 YO) is outside the corridor. The simulation curves coincide with the test curves before MCL rupture, which indicates that the material properties of the ligaments are reasonable. At the beginning, the bending moment increases with the bending angle and reaches the maximum value when the MCL is about to rupture and then the bending moment decreases sharply. The maximum bending moment of the elderly is about 110 Nm with a bending angle of 11°, while the maximum bending moment of the young is about 270 Nm with a bending angle of 18°, far greater than the elderly.

**Figure 15:**
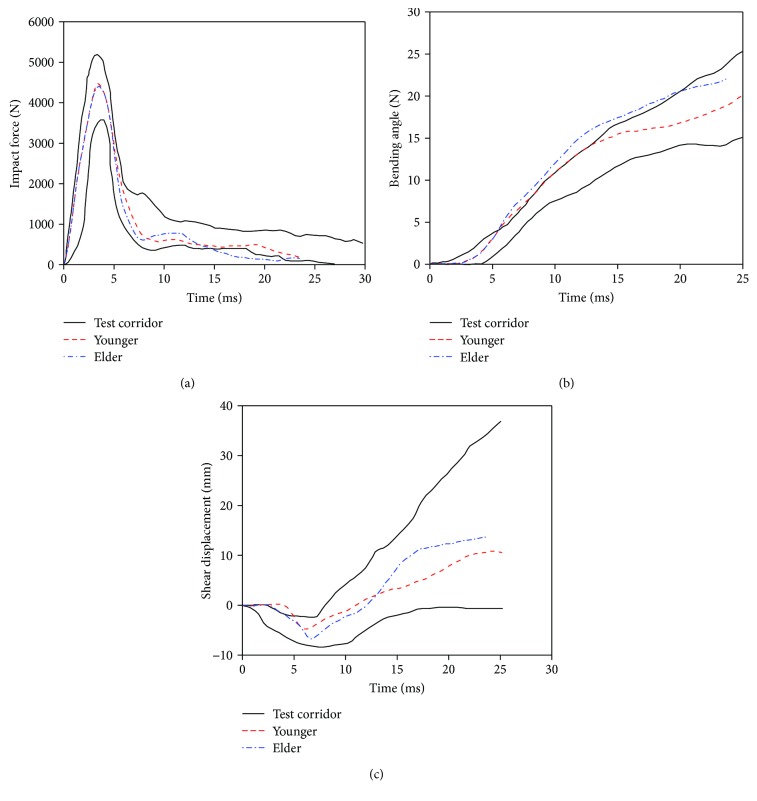
Lower extremity bending simulation results: (a) impact force, (b) knee joint bending angle, and (c) knee joint shear displacement.

The time history curves of impactor force, knee joint bending angle, and knee joint shear displacement in lower extremity bending simulation are shown in Figure 15. It indicates that the simulation results of the young and the elderly show a linear shape similar to that reported in tests and all are in the test corridor.

**Figure 16:**
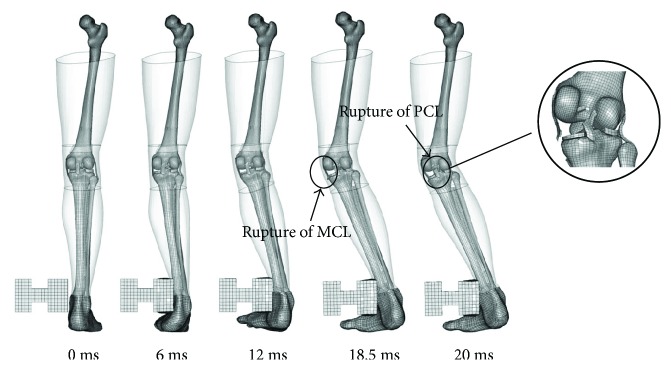
Dynamic simulation of the lower extremity simulation process.

For the impact force, there is not much difference between the simulation results of the young and the elderly. They both reach their maximum value of 4.5 kN at 4 ms. This may be due to the same kinetic energy of the impactor and the quality of the lower extremity. While the bending angle of the elderly is obviously bigger than the young beyond 10 ms, and reached 5° at 20 ms, the similar trend occurred in knee joint shear displacement curves. This is because the knee ligament strength of the elderly is much lower than the young and their ligaments usually rupture earlier in the same collision condition. For example, the MCL and PCL ruptured at 11.5 ms and 14.5 m, respectively, in the simulation. It was significantly ahead of the results of the young, which is 18.5 ms and 20 ms, respectively, and then induced larger knee bending angle and shear displacement. The kinematics of lower extremity and ligament rupture in a bending test simulation is shown in Figure 16 (take the young for example).

**Figure 17:**
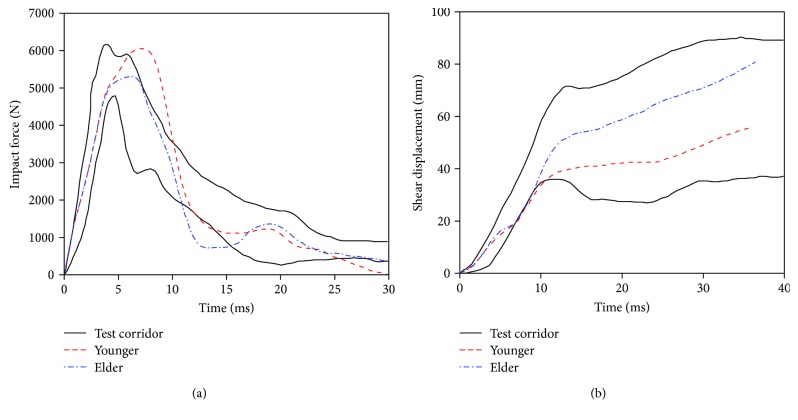
Lower extremity shear simulation results: (a) impact force and (b) knee joint shear displacement.

The time history curve of the impact force and knee joint shear displacement in the lower extremity shear simulation are shown in Figure 17. It indicates that the simulation results of the young and the elder show a similar linear shape as that reported in tests and are mostly in the test corridor, except for the impact force of the young.

**Figure 18:**
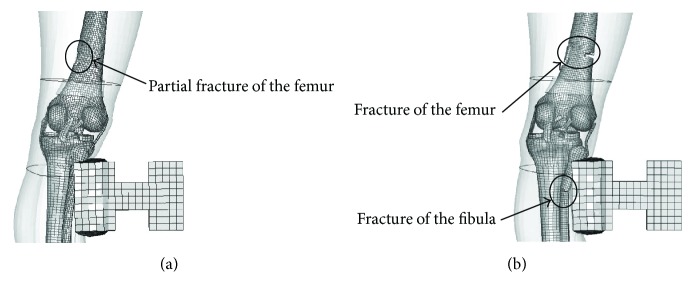
Comparison of long bone injury: (a) the young and (b) the elderly.

The peak impact force of the elderly is 5.2 kN, lower than 6.0 kN of the young, and appeared earlier at about 8 ms, while the knee joint shear displacement of the elderly is obviously larger than that of the young beyond 8 ms. It is possibly related to the elderly's femur fracture occurring at around 8 ms, resulting in the increase of rotation and lateral movement at the fracture point and the decrease of the impact force. The detailed injuries of the elderly and the young are compared in Figure 18. The young only suffered partial femur fracture, while the elder suffered full femur fracture and fibula fracture. These injuries were coordinated with that of samples 4 and 17 in the PMHS test [38].

## 4. Conclusion

In the present study, the changes of geometric and material properties of the lower extremity with aging were studied and age-specific FE models of the lower extremity for pedestrian-vehicle accident simulation were developed for 30-YO and 70-YO male pedestrian using morphing techniques. To evaluate the lower extremity response, a series of PMHS tests were simulated to validate the confidence of the models and to assess the importance of geometric and material property changes with aging. The whole age-specific FE models of pedestrian lower extremity showed numerical stability, and, in all validation simulations, the response of the young model and the elderly is different from each other. Development of age-specific FE models of the lower extremity will provide valuable tools for understanding variations in lower extremity injury patterns due to vehicle-pedestrian collision accidents across populations and in the design of new vehicles with devices for pedestrian protection.

Further study will involve the sex factor and the geometry changes of the femoral head/neck and ankle with age. These would be investigated to establish a pedestrian lower limb model with higher biofidelity. The more elaborate model and the understanding of age- and sex-specific biomechanics of the lower extremity will lead to the development of improved pedestrian protection and advancement in vehicle safety design.

## Figures and Tables

**Table 1:** The scaling ratio of long bone cross-section for a 70-YO adult.

Section position	Femur		Tibia	
	*D*_P_ (%)	*D*_M_ (%)	*D*_P_ (%)	*D*_M_ (%)
20%	1.3	4.3	5.0	12.9
35%	1.3	9.6	3.7	16.6
50%	1.8	15.2	3.3	15.7
65%	2.6	19.4	4.3	14.8
80%	4.9	19.2	5.3	11.1

**Table 2:** The long bone material properties of lower extremity model.

	Material parameters	The young (30 YO)	The elderly (70 YO)
Femoral cortical bone	Density (kg/m^3^)	2000	2000
	Elastic modulus (MPa)	16.2	13.9
	Poisson's ratio	0.3	0.3
	Yield stress	100.22	94.4
	Limit strain	0.032	0.019

Tibial cortical bone	Density (kg/m^3^)	2000	2000
	Elastic modulus (MPa)	18.3	15.7
	Poisson's ratio	0.3	0.3
	Yield stress	120.3	113.28
	Limit strain	0.034	0.020

Femur cancellous bone	Density (kg/m^3^)	1000	1000
	Elastic modulus (MPa)	752	816.4
	Poisson's ratio	0.45	0.45
	Yield stress	13.25	10.22
	Limit strain	0.134	0.134

Tibial cancellous bone	Density (kg/m^3^)	1000	1000
	Elastic modulus (MPa)	752	591.6
	Poisson's ratio	0.45	0.45
	Yield stress	11.04	8.24
	Limit strain	0.134	0.134

**Table 3:** The ligament material properties.

	Material parameters	The young (30 YO)	The elderly (70 YO)
Knee ligament	Density (kg/m^3^)	1000	1000
	Volume modulus (GPa)	4.31	3.5
	C1	34.29	22.13
	C3	1.54	0.6
	C4	152.85	147.8
	C5	836.42	695.66
	Failure strain	0.45	0.263

C1: first Mooney-Rivlin constant; C3: constant scaling of the collagen exponential stresses; C4: constant controlling rate of the rise of collagen exponential stresses; C5: modulus of straightened collagen fibers.

**Table 4:** Biomechanical cadaver tests for lower extremities.

Item	Cadaver test	Age of the PMHS (YO)
Thigh and calf	Kerrigan et al. [35]	58.5 ± 4.8/9.3
Ligaments	Dommelen et al. [35, 36]	63 ± 3.3; 53.4 ± 9.9
Knee	Bose et al. [37]	53.4 ± 9.9
The whole lower extremities	Kajzer et al. [38]	51 ± 15
